# Successful pregnancy without disease progression of radioiodine refractory papillary thyroid carcinoma: a case report

**DOI:** 10.1186/s12885-017-3717-3

**Published:** 2017-11-09

**Authors:** Yuchen Jin, Min Liu, Lingxiao Cheng, Libo Chen

**Affiliations:** Department of Nuclear Medicine, Shanghai Jiao Tong University Affiliated Sixth People’s Hospital, Yishan Rd. 600, Shanghai, 200233 People’s Republic of China

**Keywords:** Radioiodine refractory papillary thyroid carcinoma, Pregnancy, Prognosis, Thyroid stimulating hormone, Thyroglobulin

## Abstract

**Background:**

Pregnancy is an unquantifiable risk to accelerate tumor growth of papillary thyroid carcinoma (PTC), and whether pregnancy induces an unfavorable prognosis of radioiodine refractory papillary thyroid carcinoma (RR-PTC) remains unknown.

**Case presentation:**

We investigated the impact of pregnancy on the prognosis of pulmonary metastases in an RR-PTC woman via a long-term clinical follow-up and consecutive computed tomography examinations and serum tests. After a successful pregnancy, the metastatic lesions shrank with serum thyroglobulin slightly fluctuated under sustained thyroid stimulating hormone (TSH) suppression, demonstrating a favorable outcome.

**Conclusions:**

This case study indicates that metastatic RR-PTC may not be aggravated by pregnancy under TSH suppression, and pregnancy should not be contraindicated in RR-PTC patients with stable disease.

## Background

With the development of diagnostic technology, increasing number of patients was diagnosed as radioiodine refractory papillary thyroid carcinoma (RR-PTC) with relatively poor prognosis [1]. However, to date, few data can be referred to predict the outcome of RR-PTC in patients who will undergo pregnancies. To bring a conclusion, an analysis of work-flow from our database registering for radioiodine (^131^I) treatment (Jan. 2014-Dec. 2016, *n* = 876) has been made. After excluding males (*n* = 269), pathological types other than PTC (80), patients with no pregnancy history (*n* = 224), patients with pregnancy before ^131^I treatment (*n* = 276), loss of follow-up (*n* = 14), pregnant patients without evidence of metastasis (*n* = 9), miscarriage before ^131^I remnant ablation (*n* = 3), there was only one patient finally included. Herein, we describe the RR-PTC case with pulmonary metastases who underwent a complete pregnancy and documents its impact on the prognosis of the disease.

## Case presentation

**Fig. 1:**
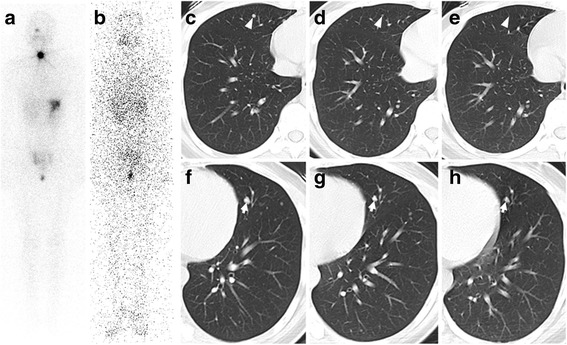
Imaging examinations. Whole body scintigraphy 3 days post the two doses of ^131^I (**a** 100 mCi for remnant ablation; **b** 200 mCi for cancer therapy) showed that there were no ^131^I–avid foci in the bilateral lungs (**b**). Diagnostic CT imaging (**c** and **f**, 3 months before getting pregnant, the maximum diameters of the largest lesions in the right and left lung were 3.75 mm and 6.66 mm, respectively; **d** and **g**, 21 months post cesarean section, the maximum diameters of the largest lesions in the right and left lung were 1.83 mm and 5.34 mm, respectively; **e** and **h**, 30 months post cesarean section, the largest lesion in the right lung was not seen and the maximum diameter of the largest lesion in the left lung was 4.88 mm) revealed shrinkage of metastatic foci without new lesions. Arrowhead, the largest lesion in the right lung; Arrow, the largest lesion in the left lung

A 26-year-old female who complained of cervical nodules was referred to our hospital in Nov. 2012. PTC was then verified by ultrasound-guided fine needle aspiration cytology and multiple pulmonary nodules were found by thoracic computed tomography (CT). The patient then received near-total thyroidectomy and lymph node dissection. In the year 2013, consecutive administrations of ^131^I were given in Jan. and Jun. for remnant ablation (3700 MBq) and treatment of pulmonary metastasis (7400 MBq). Post-ablation ^131^I whole body scan (WBS) showed only thyroid remnant uptake (Fig. 1a) and post-therapy WBS (Fig. 1b) revealed no ^131^I–avid foci. Before the second administration of ^131^I, the thyroglobulin (Tg) level under thyroid stimulating hormone (TSH) stimulation peaked to 1493 ng/mL with normal anti-Tg antibody level and stable findings of chest CT.

**Fig. 2:**
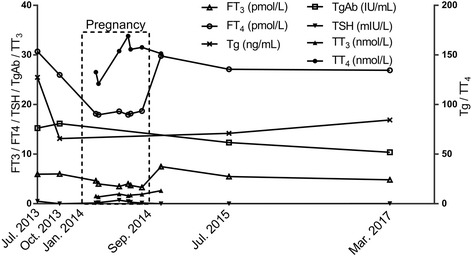
Serum tests. Serum thyroglobulin fluctuated insignificantly with sustained suppressed TSH before, during and after pregnancy. Square frame represents the period of pregnancy (Jan. 2014-Sep. 2014). FT_3_, free triiodothyronine (normal: 3.67–6.00 pmol/L); FT_4_, free thyroxine (normal: 7.50–21.10 pmol/L); TSH, thyroid stimulating hormone (normal: 0.34–5.60 mIU/L); Tg, thyroglobulin (normal: 3.50–77.00 ng/mL); TgAb, anti-Tg antibody (normal: 0.00–115.00 IU/mL); TT_3_, total triiodothyronine (normal: 1.13–2.42 nmol/L); TT_4_, total thyroxine (normal: 75.37–167.78 nmol/L)

About 7 months after the last administration of ^131^I, she got pregnant. In Sep. 2014, cesarean section was performed at 36 weeks’ gestation because of oligohydramnios. The patient delivered a healthy male infant (3 kg) with Apgar score of 10 and normal TSH level. Compared with pregestational data (baseline, Oct. 2013) (Fig. 1c and f, Fig. 2), Tg value at 10 months after cesarean section (Jul. 2015) fluctuated slightly (+8%) from 65.76 to 70.99 ng/L (Fig. 2) with evident shrinkage of pulmonary foci without new lesions demonstrated by CT (Fig. 1d and g). Thirty months (Mar. 2017) after cesarean section, serum tests revealed TSH of 0.02 mIU/L, Tg of 86.38 ng/mL and TgAb of 12.14 IU/mL, and CT examination indicated further improvement of the disease (Fig. 1e and h).

Additionally, the patient felt well before, during and after gestation at continuous TSH suppression status (0.01–0.71 mIU/L) sustained by oral administration of levothyroxine. At the time of this writing, the 32-month-old child was healthy.

## Discussion and conclusions

Pregnancy is generally an important unquantifiable risk to maternal health, which has the potential for accelerating tumor growth of PTC due to proliferative effects of fluctuating TSH, estrogen (E2) and human chorionic gonadotropin (hCG) as reported previously [2]. During pregnancy, although the fluctuation of hormones is complicated, the net effect on the prognosis of well differentiated PTC may be favorable. Some scholars believe that pregnancy does not appear to induce a poor prognosis of PTC. Most clinical outcome data also showed no difference in the rate of recurrence or long-term survival of women with well-differentiated PTC identified during pregnancy [3–5]. Sturniolo G et al. observed an association between ER-α expression and a more favorable outcome in PTC patients [6]. In addition, Rowe et al. described a favorable outcome in a pregnant woman with metastatic PTC, who gave a normal birth of a healthy male child weighing 2380 g at 34 weeks of gestation [7]. Although two doubling rises of Tg was observed in a 33-year-old woman with pT2pN1bMx PTC during her consecutive trimesters, Tg levels returned to her pre-pregnancy baseline level following each delivery [8].

Although the prognosis of RR-PTC is poorer than well differentiated individuals, patients may also live for a long time with stable disease [1]. Therefore, the impact of potential pregnancy on the prognosis of RR-PTC should be disclosed. To the best of our knowledge, this is the first RR-PTC patient with pulmonary metastases who went through a successful pregnancy without disease progression, which was assessed by both biomarker and structural modality. As is described above, clinical follow-up in combination with consecutive thoracic CT scans and laboratory analyses revealed an outcome of stable disease. Interestingly, pulmonary metastases shrank after gestation, indicating that pregnancy per se may also be a favorable factor for the prognosis of RR-DTC patients.

In summary, this case study indicates that metastatic RR-PTC may not be aggravated by pregnancy under TSH suppression, and pregnancy should not be contraindicated in RR-PTC patients with stable disease. Longer-term follow-up and more sufficient investigations are still needed.

## Abbreviations

CT: computed tomography
E2: estrogen
hCG: human chorionic gonadotropin
PTC: papillary thyroid carcinoma
RR-PTC: radioiodine refractory papillary thyroid carcinoma
TSH: thyroid stimulating hormone
